# Genetic Heterogeneity of Hepatitis E Virus (HEV) in Wild Boars in Italy

**DOI:** 10.3390/ani16142159

**Published:** 2026-07-11

**Authors:** Vittorio Sarchese, Federica Di Profio, Matteo Carnevale, Roberta Battistini, Barbara Moroni, Lisa Guardone, Serena Robetto, Riccardo Orusa, Fulvio Marsilio, Vito Martella, Barbara Di Martino

**Affiliations:** 1 Department of Veterinary Medicine, Università Degli Studi di Teramo, Località Piano D’Accio, 64100 Teramo, Italy; fdiprofio@unite.it (F.D.P.); mcarnevale@unite.it (M.C.); fmarsilio@unite.it (F.M.); bdimartino@unite.it (B.D.M.); 2 Department of S.S. Levante Ligure, Istituto Zooprofilattico Sperimentale del Piemonte, Liguria e Valle d’Aosta, Via Degli Stagnoni 96, 19100 La Spezia, Italy; roberta.battistini@izsplv.it (R.B.); lisa.guardone@unipi.it (L.G.); 3 Istituto Zooprofilattico Sperimentale del Piemonte, Liguria e Valle d’Aosta, Via Bologna 148, 10154 Torino, Italy; barbara.moroni@izsplv.it; 4 Department of Veterinary Sciences, University of Pisa, Viale Delle Piagge 2, 56124 Pisa, Italy; 5 Centro di Referenza Nazionale per le Malattie Degli Animali Selvatici (CeRMAS), Istituto Zooprofilattico Sperimentale del Piemonte, Della Liguria e Della Valle d’Aosta, 11020 Quart, Italy; serena.robetto@izsplv.it (S.R.); riccardo.orusa@izsplv.it (R.O.); 6 Department of Veterinary Medicine, Università Aldo Moro di Bari, S.p. per Casamassima Km 3, Valenzano, 70010 Bari, Italy; vito.martella@uniba.it; 7 Department of Pharmacology and Toxicology, University of Veterinary Medicine, 1078 Budapest, Hungary

**Keywords:** Hepatitis E virus, wild boar, molecular epidemiology, genetic heterogeneity, Italy

## Abstract

Hepatitis E virus (HEV) is an emerging zoonotic pathogen in Europe, where most autochthonous infections are caused by genotype 3 (HEV-3) and are due to ingestion of raw or undercooked pork meat products. Pigs and wild boars are recognized as the main reservoirs of infection. In this study, we investigated the epidemiology of HEV in wild boars from the Liguria and Piedmont regions (northwestern Italy) by screening, molecularly, a total of 98 liver samples from Liguria (n = 66, collected during the 2023–2024 hunting seasons) and Piedmont (n = 32, collected during 2022–2025) regions. HEV RNA was detected in 11 out of 98 liver samples, with an overall prevalence of 11.2%, a finding consistent with a significant viral circulation within the investigated animal population. Molecular characterization of the partial capsid coding gene was successfully achieved for eight strains revealing genetic heterogeneity and a diverse spatial distribution of the viral strains identified, with subtype 3a detected in Liguria and subtypes 3c and 3f identified in Piedmont.

## Simple Summary

Hepatitis E virus (HEV) is a leading cause of acute viral hepatitis in humans. In Europe, most infections are zoonotic, caused by HEV genotype 3 (HEV-3), which circulates in domestic pigs and wild boars. We investigated HEV in wild boars from northwestern Italy by testing 98 liver samples from Piedmont (2022–2025) and Liguria (2023–2024). HEV RNA was detected in 11 animals (11.2%). For eight of the eleven HEV positive samples, molecular analysis of the capsid gene highlighted significant genetic diversity and a distinct spatial distribution with subtype 3a found exclusively in Liguria, whereas subtypes 3c and 3f were detected in Piedmont, reflecting complex ecological dynamics.

## 1. Introduction

Hepatitis E viruses (HEVs) are recognized worldwide as causative agents of acute hepatitis in humans [1,2]. HEV is a quasi-enveloped virus [3] of approximately 27 to 34 nm in diameter classified in the family *Hepeviridae*, subfamily *Orthohepevirinae*, genus *Paslahepevirus*, and species *Paslahepevirus balayani* (*P. balayani*) [4]. The icosahedral capsid surrounds a single-stranded, positive-sense RNA genome of about 7.2 kb, with a 5′-m7G cap and a 3′-poly (A) tail, comprising three partially overlapping open reading frames (ORFs). Based on the full-length genome analysis, HEV strains within the species *P. balayani* have been assigned to at least eight distinct genotypes (HEV-1 to HEV-8) [5], with four (HEV-1 to HEV-4) mainly implicated in human disease. HEV-1 and HEV-2 are restricted to humans and cause large epidemics in developing countries due to poor hygienic measures and lack of clean drinking water. HEV-3 and HEV-4 are zoonotically transmitted to humans through consumption of undercooked and raw meat or by direct contact with infected mammals and cause sporadic and cluster cases of hepatitis E in both industrialized and developing countries [1]. While the domestic pig represents the primary livestock reservoir, the wild boar acts as the principal sylvatic reservoir, maintaining the viral cycle independently in the wild [6].

A critical factor in the ecology of HEV is active viral shedding in the feces of infected wild boars. Fecal shedding of the virus ensures significant environmental persistence, transforming natural habitats into long-term sources of exposure for other animals and humans. This risk is amplified by the geographical expansion of wild boar into peri-urban areas, which increases the risk of exposure through environmental contamination [7,8].

HEV circulation among Italian wild boar populations is characterized by pronounced regional heterogeneity, with seroprevalence (4.9–56.2%) and molecular detection rates (3.7–43.6%) varying significantly across the geographic landscape [9,10,11,12,13,14,15,16,17,18,19,20,21,22]. Furthermore, molecular surveillance reveals a high genetic diversity characterized by the co-circulation of common European subtypes (3c, 3e, and 3f) alongside the emergence of sporadic or new subtypes, such as 3l, 3m and yet unassigned HEV-3 strains [9,13,19,20,21,22]. The variegated circulation of multiple subtypes within HEV animal reservoirs is directly reflected in the strains identified in human patients. For instance, in Italy, the frequent identification of subtypes 3f, 3e, and 3c in human clinical samples [23] mirrors the viral clusters identified in local swine and wild boar populations [24].

In light of these findings, monitoring HEV circulation in high-density wild boar areas is a priority. Northwestern Italy, specifically the Piedmont and Liguria regions, represents a critical study area due to its high wild boar population density and previous reports of circulation of diverse viral strains, including the emergence of uncommon subtypes. Given the dynamic nature of viral diffusion in these areas, the aim of this study was to provide an updated genetic characterization of HEV subtypes circulating in these two regions and monitor their evolution.

## 2. Materials and Methods

### 2.1. Study Area and Sample Collection

The survey was conducted on wild boar liver samples (n = 98) collected in northwestern Italy, including Liguria (n = 66) and Piedmont (n = 32). In Liguria, sampling occurred during the 2023–2024 hunting seasons and involved the provinces of La Spezia (n = 46) and Genoa (n = 20). The animals were classified as sub-adults (≤24 months; n = 34) or adult (>24 months; n = 24), with 8 animals lacking age data. In Piedmont, 32 liver samples were collected from five provinces including Vercelli (n = 11), Novara (n = 10), Torino (n = 6), Cuneo (n = 3), and two animals with unrecorded geographic origin. Of these, 23 animals were classified as sub-adults and 5 as adults, while four animals lacked age data. Wild boars were sampled opportunistically within the framework of research projects coordinated by the Istituto Zooprofilattico Sperimentale del Piemonte, Liguria e Valle d’Aosta (IZS PLV), relying on the availability of carcasses obtained through managed hunting activities and regional diagnostic submissions during the study period. Each liver sample corresponded to a distinct wild boar, identified by a unique sample accession number. The opportunistic framework reflects the natural heterogeneity of sample availability; therefore, the dataset inherently encompasses variations in spatial distribution, sampling season, hunting pressure, local submission practices, and demographic composition.

### 2.2. Sample Processing and RNA Extraction

Liver tissues (1 g) were homogenized in 4 mL of phosphate-buffered saline (PBS, 0.15 M, pH 7.2) using 7 mL hard-tissue homogenization tubes and a Mini Bead Mill (VWR International Srl, Milan, Italy). To facilitate tissue breakdown and virion release, 0.5 mL of proteinase K (PK) at 1 mg/mL was added. The mixture was incubated at 37 °C for 3 h with shaking at 320 rpm, then heated at 60 °C for 15 min to inactivate the enzyme. Following centrifugation at 2500 rpm for 15 min at 4 °C, the supernatant was collected and used for nucleic acids extraction. Total RNA was extracted from 200 μL of tissue supernatant using the Direct-zol RNA MiniPrep Kit (Zymo Research, Irvine, CA, USA), and the eluted RNA was further purified with the OneStep PCR Inhibitor Removal Kit (Zymo Research, Irvine, CA, USA).

### 2.3. Molecular Screening

Initial molecular screening was performed by a HEV real-time reverse transcription PCR (qRT-PCR), targeting a 68 nucleotide (nt) region of the ORF3 gene highly conserved among the four major HEV genotypes [25]. For quantification, a plasmid standard was constructed by cloning the 68 bp ORF3 fragment from a wild boar strain (GenBank accession no. KU508285) [11] into a Topo TA cloning vector (Invitrogen, Milan, Italy). Viral RNA was quantified using TaqMan Fast Virus 1-Step Master Mix (Thermo Fisher Scientific, Waltham, MA, USA) in a 25 µL volume reaction including 5 µL of extracted RNA and 20 µL of reaction mix. Primers and TaqMan probe were used at concentrations of 200 nM and 100 nM, respectively. Each experiment comprised tenfold serial dilutions (10^9^ to 10^0^ copies/reaction), standardized using the first WHO international HEV RNA standard (code 6329/10). All the liver samples were also tested by using a pan-hepevirus heminested RT-PCR based on a touchdown protocol and broadly reactive primers designed to amplify a conserved 338 nt region of the RNA-dependent RNA polymerase (RdRp) gene ORF1 [26]. Samples testing positive by RT-qPCR were subjected to HEV genotyping by nested RT-PCR amplification of a partial 493 nt region of ORF2 [27]. The primers and probe used in this study are listed in Table 1.

### 2.4. Sequence and Phylogenetic Analyses

All the amplicons were purified using a QIAquick gel extraction kit (Qiagen GmbH, Hilden, Germany) and subjected to direct sequencing with BigDye Terminator Cycle chemistry and the 3730 DNA Analyser (Applied Biosystems, Foster City, CA, USA).

Sequences obtained from positive samples were then aligned with a cognate set of reference HEV genome sequences retrieved from public databases (NCBI GenBank, https://www.ncbi.nlm.nih.gov/GenBank, accessed on 25 May 2026). Basic Local Alignment Search Tool (BLAST, version 2.17.0; http://www.ncbi.nlm.nih.gov) and FASTA, version 36.3.8i, (http://www.ebi.ac.uk/fasta33, accessed on 25 May 2026) with default values were used to find homologous hits. Sequence alignment was performed using the MUSCLE multiple alignment program [28] version 3.8.425 plugin of the Geneious Prime Version 2022.2.2 (Biomatters Ltd., Auckland, New Zealand). Phylogenetic relationships were analyzed with MEGA 12 software [29], using Maximum Likelihood (ML) and assessing the statistical support for the tree topology using bootstrap resampling (1000 replicates).

### 2.5. Statistical Analysis

HEV RNA detection rates were expressed as percentages with exact binomial 95% confidence intervals (95% CI). Differences in HEV positivity between regions and age classes were assessed using two-sided Fisher’s exact test with GraphPad Prism Software version 11.0.2 (GraphPad Software, Boston, MA, USA). The significance level was set at *p* < 0.05. Given the small number of positive animals and the unbalanced opportunistic sampling design, these statistical comparisons were considered underpowered; therefore, the absence of statistically significant differences was not interpreted as evidence of equal HEV circulation across regions or age classes.

### 2.6. Sequence Submission

The ORF2 nt sequences generated in this study were deposited in GenBank under the accession numbers: PX911626-PX911633.

## 3. Results

**Figure 1:**
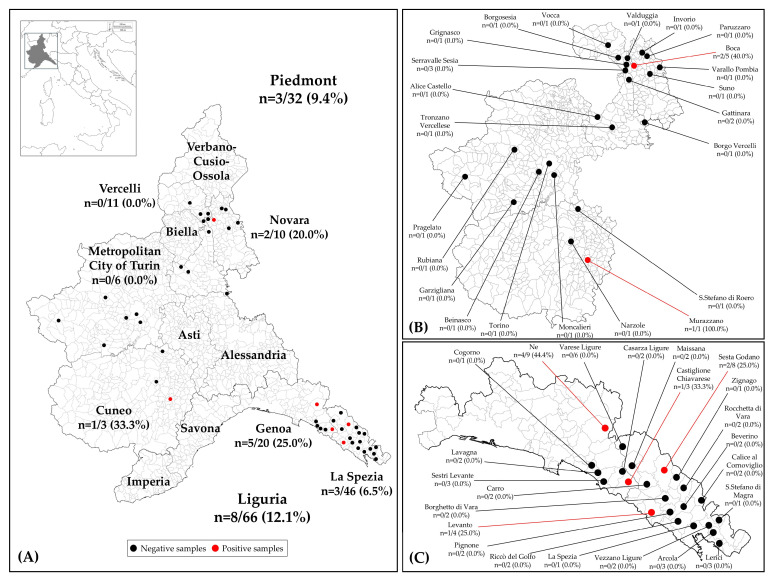
Geographic distribution of HEV-positive wild boars in Liguria and Piedmont regions. (**A**) Overall geographic distribution of sampled wild boars at regional and provincial levels; the inset shows the location of the study area in northwestern Italy. (**B**) Detailed distribution of sampling sites in Piedmont at municipal level. (**C**) Detailed distribution of sampling sites in Liguria at municipal level. Red dots indicate sampling sites where HEV-positive wild boars were detected, whereas black dots indicate sampling sites with only HEV-negative animals. For each region, province and municipality, labels report the number of HEV-positive animals over the total number tested and the corresponding positivity rate in parentheses (n = positive/tested, %). In Piedmont, two HEV-negative animals with unrecorded geographic origin are included in the regional total but not plotted at provincial/municipal level.

Out of the 98 wild boars tested, HEV RNA was detected in 11 animals with an overall prevalence of 11.2% (95% CI: 5.7–19.2). Viral load ranged from 9.1 × 10^5^ to 1.1 × 10^2^ RNA copies/5 μL of RNA template. Analyzing the geographic distribution (Figure 1) of the positivity detected, HEV RNA was found in both the regions, with molecular rates of 12.1% in Liguria (8/66; 95% CI: 5.4–22.5) and 9.4% in Piedmont (3/32; 95% CI: 2.0–25.0). The difference between regions was not statistically significant (Fisher’s exact test, *p* = 1.000). At the provincial level, HEV RNA was detected in 25.0% of wild boars from Genoa (5/20; 95% CI: 8.7–49.1), 6.5% from La Spezia (3/46; 95% CI: 1.4–17.9), 20.0% from Novara (2/10; 95% CI: 2.5–55.6), and 33.3% from Cuneo (1/3; 95% CI: 0.8–90.6). No HEV-positive samples were detected in Vercelli (0/11; 95% CI: 0.0–28.5) or in the Metropolitan City of Turin (0/6; 95% CI: 0.0–45.9). The difference between provinces was not statistically significant (Fisher’s exact test, *p* = 0.078). Among animals with available age data, HEV RNA was detected in 12.3% of sub-adults (7/57; 95% CI: 5.1–23.7) and in 10.3% of adults (3/29; 95% CI: 2.2–27.4), with no statistically significant difference between age classes (two-sided Fisher’s exact test, *p* = 1.000).

**Figure 2:**
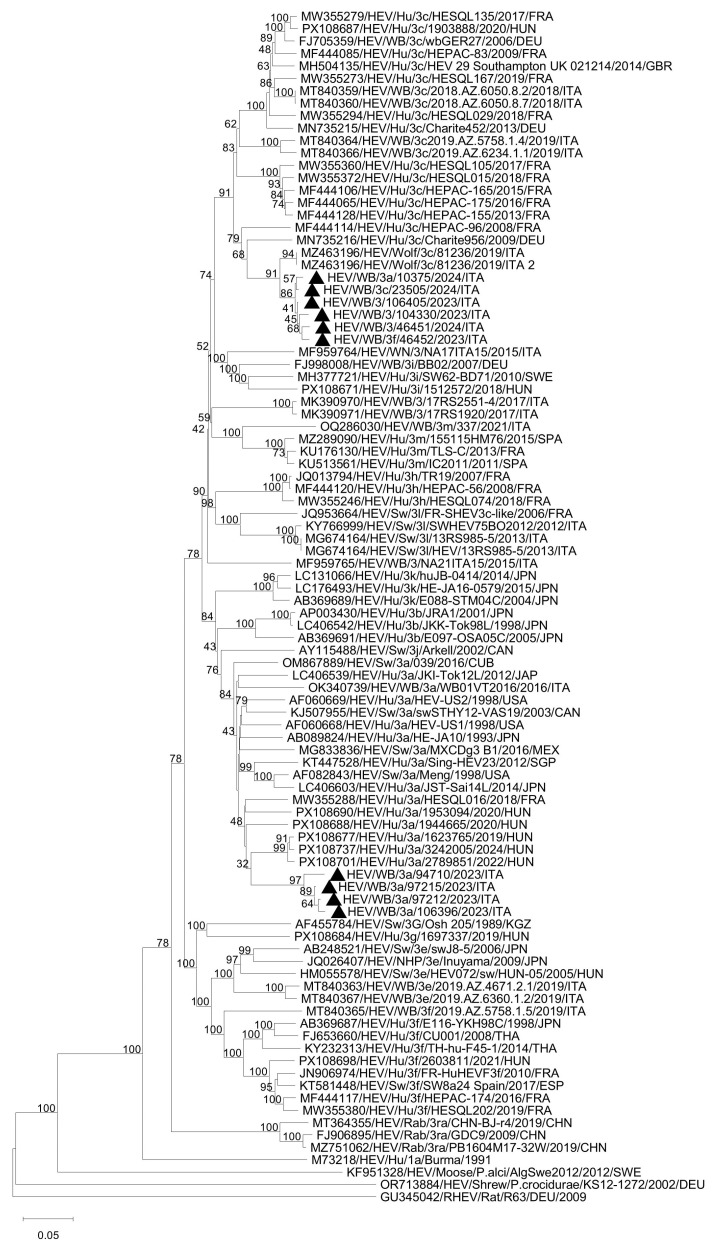
Phylogenetic tree based on a 338 bp fragment of the RNA-dependent RNA polymerase (RdRp) region within the ORF1 gene of HEV strains detected in wild boars in this study. The tree was generated using the Maximum Likelihood method based on Kimura 2-parameter model and supplying statistical support with bootstrapping of 1000 replicates. A selection of HEV sequences derived from full-length or near full-length genomes with subtype assignment was included. Black triangles indicate HEV sequences detected in this study. For the strains identified in this study (black triangle), subtype assignment, when reported, refers to the subtype inferred from the ORF2-based phylogenetic analysis, where the corresponding ORF2 fragment was available. The scale bar indicates nucleotide substitutions per site. Evolutionary analyses were conducted in MEGA12 [29]. Hu: human, Sw: swine, WB: wild boar, Rab: rabbit, NHP: non-human primates.

When re-testing all the liver samples with the pan-hepeviridae primers [26], HEV RNA was detected exclusively in the samples previously identified as positive by HEV specific qRT-PCR [25]. Specifically, the broad-range assay yielded positive results for 10 out of the 11 qRT-PCR-positive samples. In sequence analysis, pairwise comparisons of the partial RdRp amplicons from the positive samples showed nucleotide identities ranging from 81.3% to 98.5% and amino acid (aa) identities ranging from 93.1% to 100.0% among the samples. Upon BLAST and FASTA analyses, the amplicons displayed the highest identities to HEV strains classified within genotype 3, with 80.1–97.0% nt identity and 96.1–99.1% aa identity. Maximum Likelihood phylogenetic analysis of the RdRp nt fragment (Figure 2) showed that all amplified sequences grouped in the HEV genotype 3 clade.

The partial ORF2 sequence was obtained for eight strains (ID: WB/97215, WB/97212, WB/94710, WB/10375, WB/106396, WB/23505, WB/46452 and WB/94709, corresponding to GenBank Accession No. PX911626-PX911633) (Table 2).

**Figure 3:**
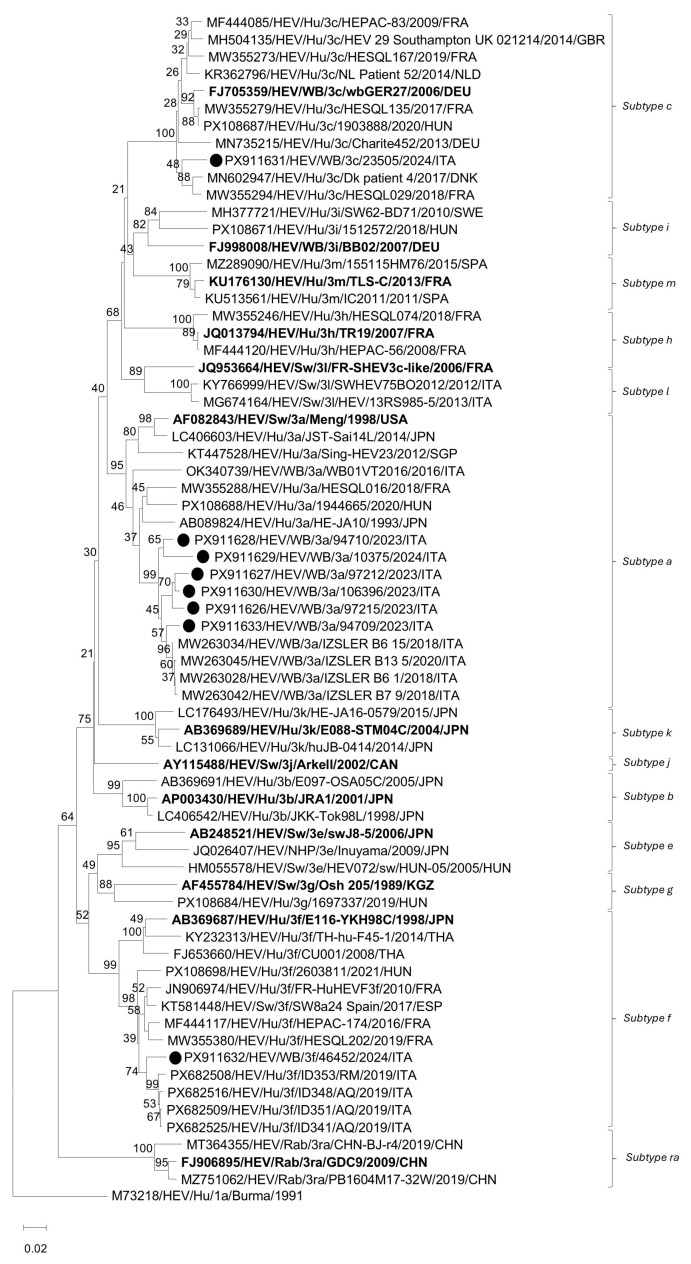
Phylogenetic tree based on 493 nt at the 5′ end of the ORF2 gene of the wild boar strains detected in this study. Tree was generated using Maximum Likelihood method based on the Tamura–Nei model and supplying statistical support with bootstrapping of 1000 replicates. A selection of partial (493 nt) nucleotide ORF2 sequences representative of each HEV-3 subtype was used for the analysis. The scale bar indicates nucleotide substitutions per site. Reference strains for each HEV-3 subtype, selected according to the proposed HEV subtype reference set reported by Smith et al. [5], are indicated in boldface. The black circles denote HEV sequences detected in this study. Evolutionary analyses were conducted in MEGA12 [29]. Hu: human, Sw: swine, WB: wild boar, Rab: rabbit, NHP: non-human primates.

Through sequence comparison in the ORF2 fragment, amplicons showed 84.3–98.6% nt and 96.7–100% aa identities to each other. Maximum Likelihood phylogenetic analysis (Figure 3) based on the 493 nt sequence of ORF2 was performed with a selection of HEV-3 strains available in databases, including reference sequences representative of each HEV-3 subtype [5]. By visual inspection of the tree, the strains identified in this study segregated into three different subtypes (Figure 3). More in detail, six amplicons (WB/97212, WB/97215, WB/94709, WB/94710, WB/10375 and WB/106396), all collected from wild boars hunted in Liguria, grouped with HEV-3a strains previously identified in Italian wild boars [17] and with sequences detected in human patients from France [30] and Hungary [31]. The nt identity within this group ranged from 91.5 to 98.1%. Furthermore, on BLAST and FASTA analyses, the 3a sequences obtained in this study, spanning a shorter available ORF2 fragment (295 nt in length), showed the highest identities (95.2–98.5% nt) to HEV strains previously detected from wild boars in the same geographical setting [19], while the nt identities to 3a sequences previously detected in Italian human patients ranged from 87.8% to 89.4% [24].

The strains WB/23505 and WB/46452, detected in liver samples from two wild boar hunted in Piedmont, grouped respectively with HEV-3c strains (90.8–96.4%) and with HEV-3f strains (87.7% to 97.2%).

The HEV sequence detected in the wild boar (WB/23505) hunted in the province of Cuneo (Piedmont region) was closely related (94.9–96.6%) with HEV-3c strains reported in an Italian wild boar in the Molise region (GenBank accession no. PV068025) and in human sera from several European countries, including France [30], the Netherlands [32], United Kingdom (GenBank accession no. MH504135) and Denmark [33]. In the phylogenetic tree, the detected sequence clustered closely with French and Danish HEV-3c strains [30,33]. Sequence analysis of the short fragment showed nucleotide identities ranging from 91.1% to 95.7%, with other HEVs 3c detected in the same host species in Umbria, Abruzzo, Liguria, and Lazio [12,13,19,20]. In addition, nucleotide sequence identities between the wild boar sequence detected in this study and HEV-3c strains detected in other animal species ranged from 90.0% to 92.5% for strains identified in sheep in Abruzzo [34], and were 90.8% for a strain detected in a wolf in Liguria [35]. Identities of 91.4–94.7% were found in other human HEV-3c strains associated with infection cases in central Italy [24,36].

The HEV-3f sequence detected in the wild boar sample WB/46452 in Piedmont (Novara) showed the highest nucleotide identity, ranging from 95.7% to 97.2% to HEV-3f strains identified in human sera collected in the Abruzzo and Lazio regions (GenBank accession nos. PX682508, PX682509, PX682516, and PX682525), clustering (94.1% to 94.8% nt identities) also with HEV-3f strains identified in swine stool samples [37] and in sera collected from human patients in France [30,38], Spain [39], and Hungary [31]. Furthermore, in the shorter fragment, sequence analysis revealed identities of 94.6–94.9% with HEV-3f strains previously identified in wild boars in Umbria and Lazio [13,20], of 90.9–97.6% with other human HEV-3f strains identified in central Italy [24], and of 89.9% with a sequence detected during a molecular investigation involving eight pig herds located in northern, central, and southern Italy [40].

## 4. Discussion

This study provides an updated assessment of the molecular epidemiology and genetic diversity of HEV in wild boar populations from northwestern Italy (Liguria and Piedmont regions).

Our findings confirm a significant circulation of HEV, with an overall prevalence of 11.2% (11/98), highlighting the role of wild boars as a persistent reservoir for HEV-3. The detection rate of 11.2% aligns with the broad range of HEV molecular prevalences reported across the Italian peninsula, which typically fluctuates between 3.7% and 43.6% depending on the region and sampling approach [9,13,15,19,20]. Positive samples were found in both of the Liguria and Piedmont regions, with similar rates of 12.1% and 9.4%, respectively. A comparable prevalence was also observed in viral detection rates between sub-adult (12.3%) and adult (10.3%) animals, suggesting that exposure to HEV may occur early in life, possibly through environmental contamination or direct contact with infected individuals. Although opportunistic sampling is a practical approach for wildlife disease surveillance, it may limit the spatial, temporal, and demographic representativeness of the dataset. Therefore, the prevalence reported in this study should not be considered representative of the entire wild boar population in northwestern Italy, but rather as an updated molecular snapshot of HEV circulation and genetic diversity within the investigated areas.

In our investigation, molecular screening was performed combining a HEV-genotypes-1–4-specific qRT-PCR [25] with a pan-hepeviridae RT-PCR [26]. Since the broad-range assay detected HEV RNA only in samples that had previously been tested positive by the qRT-PCR, and sequence analysis revealed that all detected HEV strains belonged to genotype 3, it could be suggested that no other genotypes or hepevirus species were circulating in the tested cohort.

Sequence and phylogenetic analyses of the partial ORF2 region were successful for eight of the eleven HEV-positive samples and revealed three distinct HEV-3 subtypes (3a, 3c, and 3f), confirming high HEV heterogeneity in this animal host [18]. However, because ORF2-based subtyping was not achieved for all HEV-positive samples, the subtype distribution reported here should be interpreted as referring only to the successfully characterized strains. Therefore, the co-circulation of additional HEV subtypes in the investigated wild boar populations cannot be excluded. Further molecular epidemiological investigations will be necessary to obtain a more comprehensive picture of HEV regional diversity. Notably, a geographical subtype distribution according to the origin of the samples was observed. Indeed, all six HEV strains identified in Liguria were all characterized as subtype 3a. This subtype was first reported in wild boars in Italy in 2019 in the Lazio region (Viterbo province) [16], and subsequently in 2021 in Emilia-Romagna (Parma Province) [17], in 2020 and 2022 in the Liguria region [19,41] and more recently in central Italy [42], including the Abruzzo region (GenBank accession no. PQ778493). Interestingly, there is currently no evidence of HEV-3a circulation in domestic pigs. In addition, De Sabato et al. (2020) [24] reported the first human infection with HEV-3a, identified in 2017 in a patient returning from recent travel to Albania, who also reported consumption of raw food in central Italy. However, the source of infection could not be determined [24]. The HEV-3a sequences identified in this study showed the highest identities (96.6–97.3%) to strains previously detected in wild boars from a hunting area in Parma, in the neighboring Emilia-Romagna region [17]. Nonetheless, when the analysis was restricted to a comparable short ORF2 fragment (295 nt), the detected strains showed lower nucleotide identities (87.8–89.4%) compared to the human HEV-3a [24], while the highest identities (95.2–98.5% nt) were observed with HEV-3a strains previously identified in wild boars from the same Liguria region [19]. In this region, the circulation of different HEV-3 subtypes as 3b, 3c, 3e, 3f, 3m, and unassigned subtypes has previously been reported [9,19,41,43]. Notably, subtype 3a has primarily been identified in eastern Liguria [19]. In this context, the detection of HEV-3a strains in the provinces of Genoa and La Spezia further supports the circulation of this subtype in the region. Furthermore, the high nt identity observed among Ligurian HEV-3a sequences could suggest limited genetic variability over the years, consistent with the possible persistence of closely related strains within the local wild boar population.

The two HEV strains detected in the wild boars hunted in the Piedmont region, in the provinces of Cuneo and Novara (Piedmont region), were characterized as HEV-3c and HEV-3f, respectively. A previous virological survey conducted during the 2012–2013 hunting season investigated wild boars from seven of the eight provinces of the Piedmont region [9], and identified HEV (subtypes 3f and 3e) exclusively in the province of Alessandria. Therefore, our findings expand the molecular evidence of HEV-positive wild boars to other provinces, Cuneo and Novara. Moreover, the detection of subtype 3c provides novel evidence of the circulation of this subtype in wild boars in the Piedmont region. Notably, both subtypes 3f and 3c had been previously identified in domestic pig populations investigated in the same region [44,45]. However, direct epidemiological and genetic comparisons were not possible since the ORF2 regions analyzed of the wild boar and swine strains did not overlap. Overall, HEV-3f represents the predominant subtype detected in pigs and humans in Italy, and, to a lesser extent, also in wild boars [24], whereas HEV-3c appears to be more frequently associated with wild boars and only sporadically detected in pigs [9,11,24]. Interestingly, previous phylogenetic analyses investigating possible genetic correlations among human, swine, and wild boar HEV-3 strains circulating in Italy revealed that Italian human HEV-3f and HEV-3e sequences tend to cluster more closely with animal strains from the same country. In contrast, Italian HEV-3c strains identified in wild boars and pigs appear more closely related to human HEV-3c sequences circulating in other European countries [24]. In line with these findings, the HEV-3f sequence detected in this study (GenBank accession no. PX911632) clustered closely with human strains identified in the Abruzzo (GenBank accession nos. PX682509, PX682516, and PX682525) and Lazio (GenBank accession no. PX682508) regions. However, as previously observed [24], human and animal sequences were not identical, and no direct geographical correlation could be established, since also in this case the related human strains were detected in patients from central Italy rather than from the study area. Consistent with the phylogenetic pattern previously reported for Italian HEV-3c strains [24], the HEV-3c sequence characterized in the present study (GenBank accession no. PX911631) clustered with human HEV-3c sequences from Europe, with the closest phylogenetic relatives represented by a French strain (GenBank accession no. MW355294) [30] and a Danish strain (GenBank accession no. MN602947) [33]. Although ORF2-based subtyping using a 493 nt fragment provides lower phylogenetic resolution than complete genome or longer coding-region analyses, clustering with recognized HEV subtype reference sequences supported the proposed subtype assignments. Furthermore, supplementary RdRp sequence analysis consistently confirmed the placement of these strains within HEV genotype 3.

Overall, these findings underline the importance of HEV-3 genotyping and subtyping not only for tracing viral circulation across wildlife, livestock and humans, but also for assessing the potential public health relevance of circulating strains, as increasing evidence suggests that the HEV-3 genetic background may influence clinical outcome. In particular, infections sustained by clade efg have been associated with more severe symptomatic disease, including higher hospitalization rates, increased bilirubin levels, higher serum CXCL10 concentrations and stronger liver necro-inflammatory activity [46]. Moreover, subtype 3f infections have been linked to higher viral loads, fever and more frequent hospitalization compared with subtype 3c infections [47].

## 5. Conclusions

In conclusion, this study confirms the active circulation and genetic diversity of HEV-3 in wild boar populations from northwestern Italy, confirming the role of wild boars as important reservoirs of HEV infection. The identification of multiple HEV-3 subtypes, including subtype 3a in Liguria and subtypes 3c and 3f in Piedmont, highlights distinct geographical distribution patterns and expands the current knowledge on HEV molecular epidemiology in these regions. Overall, these findings reinforce the importance of continuous molecular monitoring of HEV in wildlife to better understand viral circulation dynamics, assess zoonotic risks, and support integrated surveillance strategies.

## Figures and Tables

**Table 1:** List of primers used in this study. Nucleotide position refers to the sequence of the Gt1 subtype, a prototype strain, Burma (GenBank accession no. M73218).

Primer	Sequence (5′ to 3′)	Sense	Position	Target Gene	Reference
JHEV FW	GGTGGTTTCTGGGGTGAC	+	5262–5279	ORF3	[25]
JHEV REV	AGGGGTTGGTTGGATGAA	-	5312–5329		
JHEV P	FAM-TGATTCTCAGCCCTTCGC-BHQ	+	5284–5301		
HEV-F4228	ACYTTYTGTGCYYTITTTGGTCCITGGTT	+	4252–4280	ORF1 RNA-dependent RNA polymerase (RdRp)	[26]
HEV-R4598	GCCATGTTCCAGAYGGTGTTCCA	-	4564–4589		
HEV-R4565	CCGGGTTCRCCIGAGTGTTTCTTCCA	-	4600–4622		
HEV-orf2-fo-ch	AAYCARGGITGGCGYTCIGTIGARAC	+	5909–5934	ORF2	[27]
HEV-orf2-ro-ch	GARAAIGGRCGIGAIGGRGCIGG	-	6512–6534		
HEV-orf2-fi-ch	GAGGAGGAAGCTACCTCYGGYYTIGTIATGCTYTGYAT	+	5948–5985		
HEV-orf2-ri-ch	GGAGAAGGAGTTGGTCGRTCYTGYTCRTGYTGRTT	-	6479–6513		
HEV-orf2-fs	GAGGAGGAAGCTACCTC	+	5948–5964		
HEV-orf2-rs	GGAGAAGGAGTTGGTCG	-	6497–6513		

**Table 2:** Molecular and sequencing data of HEV-positive wild boar liver samples. The table reports wild boar ID, geographic origin, year of collection, age class, qRT-PCR Ct value, viral load, results of pan-hepevirus heminested RT-PCR and ORF2 nested RT-PCR, ORF2-based subtype assignment, and GenBank accession number. Viral load is expressed as RNA copies/5 μL of RNA template. Pos: positive, Neg: negative, n.d.: not determined.

Wild Boar ID	Province/Municipality	Year	Age Class	qRT-PCR Ct Value [25]	Viral Load	Pan-Hepevirus Heminested RT-PCR [26]	ORF2 Nested RT-PCR [27]	Subtype	GenBank Accession No.
WB/97215	Genoa/Ne	2023	Adult	18.1	9.1 × 10^5^	Pos	Pos	3a	PX911626
WB/97212	Genoa/Ne	2023	Adult	33.8	1.9 × 10^2^	Pos	Pos	3a	PX911627
WB/94710	La Spezia/Sesta Godano	2023	Adult	28.3	3.7 × 10^3^	Pos	Pos	3a	PX911628
WB/10375	La Spezia/Levanto	2024	Adult	30.5	1.1 × 10^3^	Pos	Pos	3a	PX911629
WB/104330	Genoa/Ne	2023	Adult	34.7	1.1 × 10^2^	Pos	Neg	n.d.	n.d.
WB/106396	Genoa/Ne	2023	Sub-adult	24.5	2.8 × 10^4^	Pos	Pos	3a	PX911630
WB/106405	Genoa/Castiglione Chiavarese	2023	n.d.	34.5	1.2 × 10^2^	Pos	Neg	n.d.	n.d.
WB/23505	Cuneo/Murazzano	2024	Adult	31.3	7.1 × 10^2^	Pos	Pos	3c	PX911631
WB/94709	La Spezia/Sesta Godano	2023	Adult	33.9	1.7 × 10^2^	Neg	Pos	3a	PX911633
WB/46452	Novara/Boca	2024	Sub-adult	34.1	1.5 × 10^2^	Pos	Pos	3f	PX911632
WB/46451	Novara/Boca	2024	Sub-adult	31.3	7.0 × 10^2^	Pos	Neg	n.d.	n.d.

## Data Availability

HEV ORF2 sequences generated in this study were deposited in GenBank under accession numbers PX911626-PX911633. Other data supporting the results of this study are available from the corresponding author upon reasonable request.
